# Adult Age Differences in Eye Movements During Reading: The Evidence From Chinese

**DOI:** 10.1093/geronb/gbw036

**Published:** 2016-03-31

**Authors:** Jingxin Wang, Lin Li, Sha Li, Fang Xie, Min Chang, Kevin B Paterson, Sarah J White, Victoria A McGowan

**Affiliations:** 1 Academy of Psychology and Behavior, Tianjin Normal University, Tianjin, China; 2 Department of Neuroscience, Psychology and Behaviour, University of Leicester, Leicester, UK

**Keywords:** Aging, Chinese reading, Eye movements, Word frequency

## Abstract

**Objectives:**

Substantial evidence indicates that older readers of alphabetic languages (e.g., English and German) compensate for age-related reading difficulty by employing a more risky reading strategy in which words are skipped more frequently. The effects of healthy aging on reading behavior for nonalphabetic languages, like Chinese, are largely unknown, although this would reveal the extent to which age-related changes in reading strategy are universal. Accordingly, the present research used measures of eye movements to investigate adult age differences in Chinese reading.

**Method:**

The eye movements of young (18–30 years) and older (60+ years) Chinese readers were recorded.

**Results:**

The older adults exhibited typical patterns of age-related reading difficulty. But rather than employing a more risky reading strategy compared with the younger readers, the older adults read more carefully by skipping words infrequently, making shorter forward eye movements, and fixating closer to the beginnings of two-character target words in sentences.

**Discussion:**

In contrast with the findings for alphabetic languages, older Chinese readers appear to compensate for age-related reading difficulty by employing a more careful reading strategy. Age-related changes in reading strategy therefore appear to be language specific, rather than universal, and may reflect the specific visual and linguistic requirements of the writing system.

## Introduction

Older readers (aged 60+ years) of alphabetic languages typically experience greater reading difficulty than their younger adult counterparts (aged 18–30 years). They tend to read more slowly and make more and longer fixational pauses and more regressions (backward eye movements) than younger adults (Kliegl, Grabner, Rolfs, & Engbert, 2004; McGowan, White, Jordan, & Paterson, 2014; McGowan, White, & Paterson, 2015; Paterson, McGowan, & Jordan, 2013a,2013b; Rayner, Castelhano, & Yang, 2009; Rayner, Reichle, Stroud, Williams, & Pollatsek, 2006; Rayner, Yang, Schuett, & Slattery, 2013). Moreover, they appear to have greater difficulty identifying words and often make disproportionally longer fixations on words that have a lower frequency of written usage and so are less familiar (Kliegl et al., 2004; McGowan et al., 2015; Rayner et al., 2006). Yet, compared with younger adult readers, older adult readers tend to skip words more frequently and make generally longer forward eye movements.

Based on these findings, it is argued that older adults compensate for their poorer processing of text by employing a more risky reading strategy in which they are more likely than younger adults to infer the identities of upcoming words using prior context and only partial word information (Rayner et al., 2006, 2009, 2013). Older readers will therefore skip words more often but are also more likely to misidentify words and so make more regressions to reinspect text. Moreover, in line with this general view, older adults appear to acquire less detailed visual information on each fixational pause compared with younger adults (Paterson et al., 2013a) and from a narrower region around the point of fixation (Rayner et al., 2009; Rayner, Yang, Schuett, & Slattery, 2014; but see Risse & Kliegl, 2011), especially when current processing demands are high (Payne & Stine-Morrow, 2012). However, this research has been conducted almost exclusively using alphabetic languages, and little is known about the effects of normal aging on eye movements when reading nonalphabetic languages, such as Chinese. Yet such findings will be essential for establishing the extent to which age-related changes in eye movement behavior during reading are universal.

Chinese is a major world language that is unlike the alphabetic languages that have been the focus of research to date. In particular, Chinese uses a logographic writing system in which text is printed (from left to right) as a sequence of equally spaced, box-like characters (e.g., Zang, Liversedge, Bai, & Yan, 2011). These characters are created from sequences of individual strokes (lines and dashes) and, although each character occupies the same square area of space, characters can vary in visual complexity depending on the number of strokes (Zhang, Zhang, Xue, & Yu, 2007). Moreover, while individual characters can correspond to a word, most words comprise two (and sometimes more) adjacent characters, although the boundaries between words are not demarcated using spaces or other visual cues. The visual and linguistic demands of written Chinese are therefore very different from those of alphabetic languages like English and German and so may pose particular challenges for older Chinese readers.

Research with college-aged readers shows that, as with alphabetic languages, Chinese is read by making saccadic eye movements along the lines of text, separated by brief fixational pauses (e.g., Yang & McConkie, 1999). These eye movements are sensitive to the visual and cognitive processes that underlie reading (Zang et al., 2011). In particular, effects of word frequency in Chinese reading are similar to those for alphabetic languages, and readers make shorter fixations on words that have a higher frequency of written usage (e.g., Li, Bicknell, Liu, Wei, & Rayner, 2014; Yan, Tian, Bai, & Rayner, 2006). The indication, therefore, is that decisions about *when* to move the eyes are influenced by how easily words can be identified when reading either Chinese or an alphabetic language. Readers of alphabetic languages also use a word-based strategy to guide their eye movements and target their saccades toward the center of upcoming words so that their fixations land at a *preferred viewing location* in words (e.g., McConkie, Kerr, Reddix, & Zola, 1988; Rayner, 1979). Moreover, the pattern of initial landing positions on words in these languages is very similar for young and older adult readers (Paterson et al., 2013b; Rayner et al., 2006). However, the extent to which Chinese readers employ a word-based strategy for eye guidance is less clear, and while initial landing position influences fixation duration (Li et al., 2014), and word frequency influences the probability of skipping words (Yan et al., 2006; Yang & McConkie, 1999), readers do not appear to systematically fixate specific locations within words (Li, Liu, & Rayner, 2011, 2015; Liu, Reichle, & Li, 2015; Tsai & McConkie, 2003; Wei, Li, & Pollatsek, 2013; Yang & McConkie, 1999, see also Yan, Kliegl, Richter, Nuthmann, & Shu, 2010). Chinese readers appear instead to use a processing-based strategy according to which, when the fixated word is easier to process, readers allocate more attention to the right of fixation to maximize the number of characters they can identify in the upcoming text (Li et al., 2015; Wei et al., 2013). This will influence the length of the following saccade, as a longer saccade will be needed to acquire novel character information when more upcoming characters can been identified.

Older readers are, however, likely to have much greater difficulty identifying characters than younger adult readers due to visual declines in older age (for a review, see Owsley, 2011), and this may have an important impact on their reading behavior. In particular, older adults typically have lower acuity and reduced sensitivity to the fine visual detail required for the efficient recognition of Chinese characters (e.g., Zhang et al., 2007), especially in peripheral vision (e.g., Crassini, Brown, & Bowman, 1988), and so they may find it particularly difficult to identify characters during reading. In addition, because older adults suffer more from the effects of visual crowding (Scialfa, Cordazzo, Bubric, & Lyon, 2013), characterized by the impaired recognition of visual objects when closely surrounded by similar objects (e.g., Bouma, 1971), these difficulties may be compounded for Chinese text due to the lack of spaces between words or characters (Wang, He, & Legge, 2014). However, because the evidence to date concerning when and where the eyes move during Chinese reading comes almost entirely from studies of college-aged readers, little is currently known about how reading behavior changes in older adulthood. Indeed, although several studies have been published in Chinese (e.g., Bai, Guo, Cao, Gu & Yan, 2012; Wang, Bai, Yan & Wu, 2012), most report data for older adults only. Consequently, the nature of adult age differences in eye movement behavior during Chinese reading remains unclear. In particular, it is yet to be established whether, like older readers of alphabetic languages, older Chinese readers employ a more risky reading strategy to compensate for their poorer processing of text, although this will be important for establishing whether age-related changes in eye movement behavior are universal. Such findings will also inform the ongoing development of sophisticated formal models of eye movement control, such as E-Z Reader (Reichle, Rayner, & Pollatsek, 2003) and SWIFT (Engbert, Nuthmann, Richter, & Kliegl, 2005), and extensions of these models to Chinese reading (Rayner, Li, & Pollatsek, 2007). These models provide a comprehensive account of visual and cognitive influences on oculomotor control during reading. The models have also simulated the effects of normal aging by adjusting model parameters to mimic the lower acuity and slower lexical processing of older readers (Laubrock, Kliegl, & Engbert, 2006; Rayner et al., 2006). It will therefore be important to establish whether similar mechanisms can account for age differences in eye movements for languages, such as Chinese, that have fundamentally different characteristics.

Accordingly, the present research assessed adult age differences in eye movements during Chinese reading. Young and older adult readers were presented with sentences that included a two-character target word that had a higher or lower frequency of written usage. If the older readers experience greater reading difficulty, similar to that observed for alphabetic languages, they should read more slowly and make generally more and longer fixations and more regressions and fixate the target words for longer, compared with younger readers. In addition, if decisions about when to move the eyes are guided by the lexical identification of words for both young and older readers, both age-groups should produce word frequency effects so that they have longer reading times on lower frequency target words. But if, like older readers of alphabetic languages (Kliegl et al., 2004; McGowan et al., 2015; Rayner et al., 2006), the older readers have greater difficulty identifying lower frequency words, they may produce larger word frequency effects than the younger readers with disproportionately longer fixations on the lower frequency target words. The pattern of initial landing positions on the target words will, in addition, be informative about eye guidance, and if the two age-groups produce similar patterns of initial landing positions, this will indicate they use a similar saccade-targeting strategy. But, crucially, if like older readers of alphabetic languages, the older Chinese readers compensate for their poorer processing of text by employing a more risky reading strategy, they will skip words more frequently than the younger readers and make generally longer forward eye movements. By comparison, if the strategy used by older Chinese readers differs qualitatively from that used by older readers of alphabetic languages, we may not observe the same age differences in word skipping or length of forward eye movements. Instead, the findings may reveal a pattern of age differences in eye movement behavior that reflects the specific visual and linguistic requirements of the Chinese writing system.

## Method

### Participants

Thirty-six young adults (*M* = 23.5 years, range = 18–26 years) and 36 older adults (*M* = 71.6 years, range = 61–94 years) from the Tianjin Normal University community took part in the experiment. All were native Chinese and had normal or corrected vision (20/30 or better), determined using a Tumbling E acuity chart (Taylor, 1978). Participants were selected to have at least 11 years of formal education (equivalent to senior high schooling), and all reported an interest in reading and that they read for several hours (at least) each week. Differences in vocabulary may affect reading, while studies show that age-related declines in working memory are an important component of adult age differences in reading (e.g., Payne et al., 2014). Tests of vocabulary and working memory capabilities were therefore conducted for 20 young and 20 older adults using the Vocabulary Knowledge Test from the WAIS-III Chinese version (Wechsler, Chen, & Chen, 2002) and WAIS-III Digit Span subtest (Wechsler, 1997). Vocabulary scores were similar for the young (*M* = 13.5, *SD* = 1.5) and older adults, *M* = 12.8, *SD* = 1.5; *t*(38) = 1.49, *p* > .1. However, as is typical for these age-groups (Ryan, Sattler, & Lopez, 2000), scores for the digit span task were lower for the older adults (*M* = 11.7, *SD* = 2.0) than the young adults, *M* = 15.0, *SD* = 2.0; *t*(38) = 5.30, *p* < .001. These scores refer to raw scores, not the size of the digit span.

### Materials and Design

**Figure 1.**
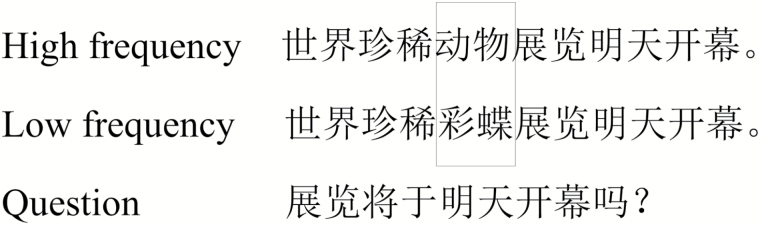
An example sentence in each condition and the accompanying comprehension question. The target words are shown within a box but were presented normally during the experiment. The sentences translate as “The world rare animal/butterfly exhibition will be held tomorrow.” The question translates as “Will the exhibition be tomorrow?”

Stimuli consisted of 40 sentence frames from Yan, Wang, and Bai (2007) that included an interchangeable two-character target word that was high (*M* = 194, *SD* = 209) or low, *M* = 12, *SD* = 8; *t*(39) = 5.56, *p* < .001, in frequency, according to the Modern Chinese Corpus (see Figure 1). These words were matched for number of strokes, for both the first character, high frequency: *M* = 7.8, *SD* = 3.36; low frequency: *M* = 6.9, *SD* = 2.57; *t*(39) < 1.5, and second character, high frequency: *M* = 7.6, *SD* = 3.89; low frequency: *M* = 7.8, *SD* = 3.69; *t*(39) < 1. A cloze task with 20 participants (who did not participate in the experiment) showed that high- and low-frequency words were equally predictable in the sentence frames, high frequency: *M* = 5.0% words guessed correctly and low frequency: *M* = 7.8% words guessed correctly, *t*(39) < 1. Sentences were 14–16 characters (*M* = 15.0) long, and target words were located near the middle of each sentence. Sentence frame and target word combinations were divided into 2 lists, each containing all 40 frames and an equal number of high- and low-frequency words, preceded by four practice sentences. Eighteen participants from each age-group were randomly allocated to each list. Accordingly, a mixed experimental design was used with the between-participants factor age-group (young adult and older adult) and within-participants factor word frequency (higher and lower). The primary dependent variables were reading times and measures of eye movements during reading.

### Apparatus and Procedure

An EyeLink 1000 eye tracker recorded right-eye gaze location every millisecond (viewing was binocular). This system has high spatial (<.01° RMS) and temporal (1,000 Hz) resolution. Sentences were displayed in Song font in black-on-white text. Each character subtended approximately .75° horizontally and so was of a typical size for reading. Participants were instructed to read normally and for comprehension. At the start of the experiment, a 3-point horizontal calibration procedure was conducted across the same line as text was presented (ensuring .30° or better accuracy for all participants and so accurate to within half a character). Calibration accuracy was checked before each trial, and the eye tracker was recalibrated if accuracy was below this criterion. At the start of each trial, a fixation square equal in size to one character was presented on the left side of the screen. Once this was fixated, a sentence was presented with the first character replacing the square. Participants pressed a response key once they finished reading each sentence. The sentence was then replaced by a comprehension question on 30% of trials to which participants responded by pressing a key.

## Results

Accuracy in answering comprehension questions (analyzed using linear mixed-effects models [LMEMs], see subsequently) was high for all participants but higher for young (*M* = 95%) than older (*M* = 89%) adults (β = .93, *SE* = .31, *z* = 2.99). Thus, while the older readers comprehended the sentences well, they did so less well than the younger readers. Following standard procedures, short (<80ms) and long (>1,200ms) fixations were deleted (affecting 5.1% of fixations). This affected similar proportions of short and long fixations for the young and older adults. Trials were excluded if tracker loss or error occurred or reading measures exceeded 2.5 *SD* of each participant’s mean.

Several standard sentence-level measures are reported: sentence reading time, average fixation duration, number of fixations, number of regressions, and progressive saccade length (see Table 1). We also report two novel sentence-level measures. *First-pass sentence reading time* is the sum of all first-pass fixations, that is, fixations from the first time a word is encountered before a saccade to another word, excluding words that initially were skipped. *Sentence rereading time* is the sum of fixations on words after first-pass, including fixations on words that initially were skipped. In addition, standard word-level measures are reported for the target words: word skipping, first fixation duration, gaze duration, total reading time, refixation probability, regressions-in, initial landing position, and the launch site of the saccade prior to the first fixation on the target word (see Table 3). Except for total reading time and regressions-in, these are measures of first-pass reading and so informative about the initial processing of words. We calculated the initial landing positions of fixations based on the width of the two-character target words, reported as the percentage distance in from the word’s left boundary to the initial fixation position. The launch site of the saccade prior to this fixation was calculated as the distance from the word’s left boundary to the position of the previous fixation.

**Table 1.** Means for the Sentence-Level Measures

	Young	Older	AE
Sentence reading time (ms)	3,158 (161)	5,047 (211)	1,889
Average fixation duration (ms)	258 (6)	284 (7)	26
Number of fixations	11.3 (0.4)	16.2 (0.6)	4.9
Number of regressions	2.5 (0.2)	4.1 (0.3)	1.6
First-pass sentence reading time (ms)	1,947 (89)	2,800 (129)	853
Rereading time (ms)	934 (108)	1619 (126)	685
Progressive saccade length (characters)	2.0 (0.1)	1.7 (0.1)	-0.3

*Note.* Standard errors are reported in parentheses. AE = age effect (mean of the older adults minus the mean of the young adults). Sentence reading time is the time from the onset of a sentence display until the participant presses a response key to indicate they have completed reading the sentence. Average fixation duration is the mean length of all fixations. Number of fixations is the count of these fixations, and number of regressions is the count of all backward eye movements, including regressions preceding refixation of a character. First-pass sentence reading time is the sum of all first-pass fixations, and sentence rereading time is the sum of fixations on words after first pass. Progressive saccade length is the average length, in characters, of forward eye movements.

Data were analyzed by LMEMs (Baayen, Davidson, & Bates, 2008) using R (R Development Core Team, 2012) and the lme4 package (Bates, Maechler, & Bolker, 2011). This approach has advantages over approaches based on traditional analyses of variance by simultaneously taking account of the separate sources of error variance associated with participants and stimuli in the same statistical model. For binomial variables, generalized LMEMs were conducted with the Laplace approximation. A maximal random effects structure was used (Barr, Levy, Scheepers, & Tily, 2013), with participants and items specified as crossed random effects. For sentence-level measures, age-group was a fixed factor, and for target word measures, age-group, word frequency, and their interaction were fixed factors. Contrasts of main effects and contrasts to examine the interactions between age-group and word frequency were defined using sliding contrasts (the contr.sdif function) in the MASS package (Venables & Ripley, 2002). As there were only two levels of each variable, these contrasts produced effect coding for the main effects and so were equivalent to other methods such as contr.sum. Following convention, *t*/*z* values greater than 2 were considered significant. Data for continuous variables were log transformed with the exception of number of regressions, sentence rereading time, and initial landing positions, for which log transformation did not improve fit. Models are first reported for all participants without covariates and then separately for participants for whom vocabulary and digit span was assessed, with these scores included as predictor variables. Models that included a variable identifying participants who provided vocabulary and digit span measures produced no interactions involving this variable (all *t*s < 1.8), indicating that these participants behaved similarly to the other participants.

### Sentence-Level Analyses

Table 1 shows the sentence-level means, and Table 2 summarizes statistical effects. The older adults read more slowly than the young adults and made more and longer fixations and more regressions. These findings resonate with the findings from alphabetic languages (Kliegl et al., 2004; McGowan et al., 2014, 2015; Paterson et al., 2013b; Rayner et al., 2006). In addition, compared with the young adults, the older adults had longer first-pass sentence reading times and longer sentence rereading times, indicating that they read more slowly during both initial sentence reading and later rereading. However, in contrast with previous findings for alphabetic languages, the older adults made shorter rather than longer progressive saccades compared with the young adults. Therefore, rather than exhibiting risky reading, the older readers moved their eyes more cautiously.

**Table 2.** Statistical Effects for Sentence-Level Eye Movement Measures

		Sentence reading time	Average fixation duration	Number of fixations	Number of regressions	First-pass sentence reading time	Sentence rereading time	Progressive saccade length
Intercept	β	8.22	5.59	2.55	3.30	7.69	1279.29	0.57
	*SE*	0.04	0.02	0.03	0.20	0.04	90.20	0.03
	*t*	225.8	325.5	83.53	16.92	217.42	14.20	21.85
Age-groups	β	0.50	0.10	0.37	1.61	0.36	687.19	0.17
	*SE*	0.07	0.03	0.05	0.36	0.07	166.03	0.05
	*t*	7.73*	2.90*	6.91*	4.42*	5.44*	4.14*	3.41*

*Note.* Asterisks are used to indicate statistically significant fixed-factor effects (*t* > 2).

### Target Word Analyses

Table 3 shows the target word means, and Table 4 summarizes statistical effects. Compared with the young adults, the older adults made longer initial fixations on words (first fixation durations and gaze durations) and had longer total reading times, consistent with the findings for alphabetic languages (Kliegl et al., 2004; Paterson et al., 2013b, Rayner et al., 2006). These findings further indicate that the older adults experienced greater reading difficulty. The older adults were also more likely to refixate target words. However, in contrast with prior findings for alphabetic languages, the older adults skipped words less frequently than the young adults. Together the word skipping and refixation effects, and the sentence-level finding that older adults made shorter progressive saccades, suggest that the older adults did not engage in risky reading. Instead, the older readers appear to use a more careful strategy in which they skip words infrequently, refixate words more often, and generally make more cautious forward eye movements than their younger counterparts.

**Table 3.** Target Word-Level Eye Movement Measures

	Young adults		Older adults		AE	FE
	High	Low	High	Low		
Word skipping (%)	13.9 (2.7)	10.1 (1.6)	6.5 (1.4)	4.5 (1.1)	−6.5	−2.9
First fixation duration (ms)	240 (7)	255 (8)	278 (8)	294 (9)	38	15
Gaze duration (ms)	297 (12)	329 (15)	385 (17)	451 (21)	105	49
Total reading time (ms)	453 (25)	524 (29)	554 (24)	721 (42)	149	118
Regressions-in probability (%)	26.1 (2.4)	28.3 (2.8)	25.4 (2.1)	31.7 (2.2)	1.3	4.2
Refixation probability (%)	22.2 (2.6)	26.9 (2.7)	34.7 (3.3)	42.1 (3.0)	13.9	6.1
Landing position (% into word)	44.4 (1.2)	43.9 (1.4)	38.4 (1.4)	36.9 (1.1)	−6.5	1.0
Launch site (characters)	1.3 (0.1)	1.2 (0.1)	1.0 (0.1)	0.9 (0.1)	−0.3	−0.1

*Notes.* AE = age effect (mean of the older adults minus the mean of the young adults). FE = frequency effect (mean of the low frequency items minus the mean of the high frequency items). Standard errors are shown in parentheses. Word skipping is the probability of a word not receiving a fixation during first pass. First fixation duration is the duration of the first first-pass fixation on a word. Gaze duration is the sum of all first-pass fixations on a word, and refixation probability is the probability of fixating a word more than once during first-pass reading. Total reading time is the sum of all fixations on a word. Regressions-in is the probability of a backward movement to reinspect a word. Initial landing position refers to the location of the first first-pass fixation on a word, reported as the percentage distance in from the left boundary of the target word. Launch site is the distance from the right boundary of the target word of the fixation prior to the first fixation on the target word.

**Table 4.** Statistical Effects for Target Word-Level Eye Movement Measures

		Word skipping	First fixation duration	Gaze duration	Total reading time	Regressions-in	Refixation probability	Landing position	Launch site
Intercept	β	3.07	5.52	5.76	6.15	1.05	0.90	0.41	1.08
	*SE*	0.19	0.02	0.03	0.04	0.10	0.11	0.01	0.05
	*t/z*	16.52	252.27	185.57	145.54	10.68	8.29	52.36	19.92
Age-group	β	0.96	0.14	0.26	0.27	0.09	0.70	0.06	0.28
	*SE*	0.36	0.04	0.05	0.06	0.15	0.19	0.01	0.11
	*t/z*	2.69*	3.39*	4.88*	4.17*	0.59	3.62*	4.41*	2.65*
Word frequency	β	0.12	0.05	0.12	0.18	0.20	0.37	0.01	0.05
	*SE*	0.16	0.02	0.03	0.04	0.10	0.11	0.01	0.03
	*t/z*	0.72	2.96*	4.49*	5.13*	2.03*	3.38*	0.62	1.55
Age-Groups × Word Frequency	β	0.05	0.01	0.05	0.08	0.23	0.08	0.01	0.04
	*SE*	0.32	0.03	0.04	0.04	0.19	0.18	0.02	0.06
	*t/z*	0.15	0.27	1.43	1.84	1.20	0.43	0.60	0.59

*Note.* Asterisks are used to indicate statistically significant fixed-factor effects (*t/z* > 2).

First fixation durations, gaze durations, and total reading times were shorter; refixation and regression rates were lower; and skipping probabilities were numerically greater for the higher frequency words. These effects complement the findings from previous Chinese studies with young adults (Li et al., 2014; Yan et al., 2006). No significant interactions between age-group and word frequency were observed in first fixation duration or gaze duration. Thus, the effects of word frequency were broadly similar for the two age-groups during first-pass reading. (Interaction effects are notoriously difficult to examine, Wagenmakers, Krypotos, Criss, & Iverson, 2012. One recommendation is to examine the effects of transformations on an interaction. Our analyses showed no interactive effects of age-group and word frequency for both log-transformed data and reciprocal-transformed data.) For total reading time, which provides an indication of later processing, the word frequency effect was numerically larger for older (167ms) than younger (71ms) adults. No interactions with age-group were observed for refixation probabilities, regressions-in, or word skipping. Word frequency effects were therefore broadly similar for the two age-groups, especially during first-pass reading.

### Initial Landing Positions

Table 3 shows mean initial landing positions and launch sites for the two-character target words, and Table 4 summarizes statistical effects. Initial fixations tended to land on the first character of words, consistent with previous findings for young adults (Li et al., 2011; Yan et al., 2010). However, fixations landed less far in from the left boundary of the target words for the older (37.6%) than younger (44.2%) adults. In addition, the launch sites of the saccades that produced these fixations were less far from the beginning of the target words for the older than younger adults. The magnitude of both effects was small. However, landing positions closer to the beginning of words are associated with higher refixation probabilities (Yan et al., 2010), and more cautious reading (Paterson & Jordan, 2010) and closer launch sites are consistent with more careful reading. Accordingly, these age differences in eye guidance are consistent with other indications that the older adults read more carefully. The effects contrast with the findings from alphabetic languages showing similar patterns of landing positions for young and older adult readers (Rayner et al., 2006; Paterson et al., 2013b).

### Effects of Vocabulary and Digit Span

Additional analyses were conducted for the participants whose vocabulary and digit span were assessed. Base models were constructed using the same variables as analyses for all participants. Model comparison were then used to determine whether adding vocabulary or digit span as additional predictor variables improved model fit (Baayen et al., 2008). The base models replicated the age effects reported above for all measures except average fixation duration. Including vocabulary as a predictor variable improved model fit only for sentence rereading time, χ^2^(1) = 4.36, *p* < .05, and regressions-in, χ^2^(1) = 4.12, *p* < .05]. The model for sentence rereading time showed an effect of vocabulary (β = 172.29, *SE* = 84.51, *t* = 2.94) in addition to an effect of age-group (β =826.20, *SE* = 252.26, *t* = 3.23) but no interaction (β = −15.48, *SE* = 169.02, *t* = .10). The model for regressions-in showed a main effect of vocabulary only (β = .14, *SE* = .07, *t* = 2.09) and no interaction (β = .23, *SE* = .23, *t* = .37). The indication, therefore, is that lower vocabulary was associated with more regressions to target words and more rereading but that this influence occurred independently of the effects of age-group. Including digit span as a predictor variable improved model fit for first-pass reading times for sentences, χ^2^(1) = 4.44, *p* < .05], and first fixation durations, χ^2^(1) = 4.63, *p* < .05, and gaze durations, χ^2^(1) = 3.75, *p* = .053, for target words. Lower digit spans were associated with longer first-pass reading times (β = .05, *SE* = .03, *t* = 2.08) and longer first fixation durations (β = .03, *SE* = .01, *t* = 2.14) but not gaze durations (β = .04, *SE* = .02, *t* = 1.91), and effects of digit span did not interact with age-group (all *t*s < 1.57). Therefore, working memory effects were independent of effects of age-group. Stronger effects of working memory in other studies (e.g., Payne et al., 2014) may be because these studies focused on aspects of language processing (e.g., resolution of syntactic ambiguities) that have a large working memory component.

## Discussion

The present study provides novel evidence concerning the eye movements of older Chinese readers. Compared with younger readers, the older adults read more slowly, made more and longer fixations, more regressions, and fixated target words for longer. These findings add to the growing evidence that older adults experience greater reading difficulty (Kliegl et al., 2004; McGowan et al., 2014, 2015; Paterson et al., 2013b; Rayner et al., 2006, 2009, 2013) and now show this age-related difficulty extends to Chinese reading.

Both age-groups also produced robust word frequency effects, with shorter fixations for higher frequency words. This effect emerged early in the eye movement record, in gaze durations for target words, and so it seems clear that both age-groups were sensitive to the lexical characteristics of words during first-pass reading. This replicates previous findings for young adults showing word frequency effects for early reading measures during Chinese reading (Li et al., 2014; Yan et al., 2006) and now extends these findings to older readers. As this word frequency effect was of similar size for the two age-groups, especially during first-pass reading, it appears that despite their overall slower reading, older readers had no particular difficulty identifying lower frequency words. Previous indications of larger frequency effects for older readers of alphabetic languages have been taken to show older adults have greater difficulty identifying lower frequency words (Kliegl et al., 2004; McGowan et al., 2015; Rayner et al., 2006), although such effects have not been observed in studies of isolated word recognition (Allen, Madden, Weber, & Groth, 1993; Tainturier, Tremblay, & Lecours, 1989). As little is known about the effects of aging on isolated word recognition in Chinese, it will be important to establish whether word frequency effects are also preserved for older Chinese readers in these paradigms.

It was of particular importance for the present research that, by contrast with the findings for alphabetic languages, the older adults did not skip words more frequently than the young adults and made shorter, rather than longer, forward eye movements. Together these findings reveal that, rather than engaging in risky reading to compensate for greater reading difficulty, older Chinese readers employ a more careful strategy in which they skip words infrequently, refixate words more often, and make more cautious forward movements. The pattern of initial landing positions and launch sites supported this view. Consistent with previous research with young adults (Li et al., 2011; Yan et al., 2010), landing positions tended to be on the first character of the two-character target words for both age-groups. But, for the older adults, these fixations landed less far into target words and launch sites were closer to the beginning of target words, and so it appears the older readers targeted their saccades more cautiously.

This more careful reading strategy may be a consequence of the visual and linguistic requirements of written Chinese. In particular, because Chinese is naturally unspaced and lacks visual cues to word boundaries, readers must acquire information about the identities of characters to the right of fixation (in parafoveal vision) to establish word boundaries and make a head start in processing words. There is growing evidence that Chinese readers do not use a word-based strategy for eye guidance and do not target their saccades toward the center of upcoming words (Li et al., 2011, 2015; Tsai & McConkie, 2003; Wei et al., 2013; Yang & McConkie, 1999), and the landing position effects in the present experiment are consistent with this view. Current accounts of oculomotor control during Chinese reading hold that readers instead employ a specific processing-based strategy such that, when the presently fixated word is easier to process, they allocate more attentional resources to the processing of characters to the right of fixation (Li et al., 2015; Wei et al., 2013). This will determine the length of the following forward saccade, as when the fixated word is easier to process, readers can identify more characters to the right of fixation, and so make a longer forward saccade to acquire novel character information.

This processing-based strategy emphasizes the central importance of parafoveal processing in Chinese reading. However, the account is based on data from young adults, and the extent to which older readers use a similar strategy remains to be determined. Indeed, it is well established that changes in visual abilities that occur naturally with older age (e.g., see Owsley, 2011) produce lower acuity and reduced sensitivity to visual detail, especially in more peripheral vision (e.g., Crassini et al., 1988) that may limit parafoveal processing. Older adults also tend to experience increased effects of visual crowding and so have reduced the ability to recognize visual objects in clutter (Scialfa et al., 2013), and this too may impair character identification particularly when text is unspaced (e.g., see Wang et al., 2014, Zhang, Zhang, Xue, Liu, & Yu, 2009). Accordingly, older readers may skip words less often and make shorter forward saccades, because they are less able to parafoveally process character identities. However, the actual influence of these visual changes on reading has not been investigated, and so it will be crucial for future research to more fully reveal the effects of aging on parafoveal processing during Chinese reading.

It will also be important to establish the extent to which other factors, including intergenerational differences in cultural and reading experiences, and the periodic modernization of Chinese characters, contribute to age differences in reading. For instance, if older readers have less rich reading experiences or lack fluency with modern characters, this might provide an alternative explanation for any difficulties they experience. Crucially, however, the indication is that this was not an important consideration for the present research. In particular, the effects of word frequency during first-pass reading, considered a hallmark of normal word identification process during reading, were broadly similar for the young and older readers, and infrequent words posed no particular problems for the older readers. In addition, the older readers showed high levels of comprehension accuracy. Consequently, the indication is that although the older adults read more slowly, they nevertheless were able to comprehend text well. Indeed, the more careful reading strategy employed by older Chinese readers may be an adaptive response to changes in visual and reading abilities in later life that helps readers identify words most efficiently.

The present findings contribute to general efforts to understand the effects of healthy aging on reading comprehension (Gordon, Lowder, & Hoedemaker, 2015). This research is largely focused on the reading of alphabetic languages, and the present study highlights the importance of extending this work to logographic languages like Chinese. The present findings will also aid the further development of formal models of eye movement control during reading. Current models (E-Z Reader and SWIFT) differ in their core theoretical assumptions (e.g., serial sequential lexical processing vs. parallel lexical processing). However, both have been largely successful in accounting for the effects of aging on eye movements when reading alphabetic languages (Laubrock et al., 2006; Rayner et al., 2006). Within E-Z Reader, this was achieved by adjusting parameters to slow lexical processing and increase the effects of word frequency (Rayner et al., 2006). Effects of aging have also been simulated in SWIFT, primarily by adjusting parameters to produce generally slower rates of processing (Laubrock et al., 2006), although this did not reproduce increases in word skipping for older readers. It will be essential to determine whether the models can also account for age differences in oculomotor control during Chinese reading. Such models may also help establish the mechanisms underlying these age differences by, for example, revealing if the reading strategy used by older Chinese readers is a consequence of reduced parafoveal processing.

In sum, the present research provides fresh insights into adult age differences in eye movement control during reading by revealing that older Chinese readers employ a more careful strategy in which they move their eyes more cautiously along the lines of text to compensate for the greater difficulty they experience. To what extent this strategy reflects the specific visual and linguistic requirements of the Chinese writing system remains to be determined, but the clear indication is that age-related changes in reading strategy may be language specific rather than universal.

## Funding

The work reported in this article was supported by an Experimental Psychology Society study visit grant and Economic and Social Research Council grant ES/L010836/1.
